# Comparison of Antioxidant Activity and Total Phenol Contents of some Date Seed Varieties from Iran

**Published:** 2010

**Authors:** Mohammad Reza Shams Ardekani, Mahnaz Khanavi, Mannan Hajimahmoodi, Maryam Jahangiri, Abbas Hadjiakhoondi

**Affiliations:** a ***Department of Pharmacognosy and Medicinal Plants Research Center, Faculty of Pharmacy, Tehran University of Medical Sciences, Tehran, Iran.***; b ***Department of Traditional Pharmacy, School of Traditional Medicine, Tehran University of Medical Sciences, Tehran, Iran.***; c ***Department of Drug and Food Control, Faculty of Pharmacy, Tehran University of Medical Sciences, Tehran, Iran.***

**Keywords:** Antioxidant, Total phenol, *Phoenix dactyliferea *L., Seed, Date

## Abstract

The genus *Phoenix *is one of the most widely cultivated groups of palms around the world. The aim of this study was to determine the antioxidant activity and total phenolic compounds of 14 different varieties of date palm (*Phoenix dactylifera *L.*, *Arecaceae) seed extracts with 5 solvents [water, methanol, methanol (50%), DMSO, and water: methanol: acetone: formic acid (20:40:40:0.1)]. Ferric reducing antioxidant power assay and Folin-Ciocalteu reagent was used for determination of the antioxidant effect and phenolic content of date seeds. DMSO extract of the “Zahedi” variety had the highest antioxidant effect (37.42 mmol/100 g dry plant) and total phenolic content (3541 mg /100 g dry plant) among these 14 varieties and 5 solvents. There was a significant correlation between the total phenolic content and antioxidant activity (R^2^ = 0.791, P < 0.001) of the “Zahedi” variety DMSO extract, which can indicates that polyphenols are the main antioxidants.

Iranian date palm seed has a relatively high antioxidant activity due to contribution of phenolic compounds. The present study showed that the Iranian date seeds are strong radical scavengers and can be considered as a good source of natural antioxidants for medicinal and commercial uses.

## Introduction

The date palm (*Phoenix dactylifera *L.*, *Arecaceae) is one of mankind’s oldest cultivated plants (1). It is highly popular worldwide, particularly in the Middle East and North Africa (2). Iran produced 918000 metric tons of dates in 2006, contributing about 21% to the total global production (3).

Date has been used as food for 6000 years (1). It could be used for generations to come due to its remarkable nutritional, health and economic value in addition to its aesthetic and environmental benefits (4). The fruit of the date palm is well known as a staple food which is composed of a fleshy pericarp and seed (5). The date seeds (pits) constitue approximately 10% of the fruits (6). A date is a high-energy food being high in carbohydrates (70-80%) and low fats and proteins, and a good source of vitamin, calcium, magnesium, phosphorous, zink, iron, potassium and iodine (7-10). Most of the carbohydrates in dates are in the form of fructose and glucose, which are easily absorbed by the human body (9). 

Also, the date seeds contain high percentage of carbohydrate (81.0-83.1%), protein (5.17-5.56%), oil (10.19-12.67%), ash (1.12-1.15%) and oleic acid (41.3-47.7%) (5). 

Date fruits are listed in folk remedies for treatment of various infectious diseases, atherosclerosis, diabetes, hypertension and cancer (7, 11, 12). There are some reports of the enhancing effect of date fruit on haemagglutinating antibody titers, plaque-forming cell counts in spleen, and macrophage migration index as an index of cell-mediated immunity (7).

A common denominator in pathogenesis of most of the chronic diseases is the involvement of oxidative stress, related to the production by all aerobic organisms of reactive oxygen and nitrogen species, including free radicals (13-18). In addition to having a role in intra and extra cellular signaling, these reactive molecular species may initiate damaging biochemical reactions (17-19). In response to such damages, a complex antioxidant defense has been developed, and dietary antioxidants comprise an important role in this defense (20-23). 

Antioxidant activity is closely related to the phenolic content of plants (3). Because synthetic antioxidants such as butylated hydroxytoluene or butylated hydroxyanysol could promote cancer development in rats and the fact that consumers are much interested in natural food additives, herbal phenolic compounds and other natural antioxidants are extremely desirable (24, 25). Some of these compounds were found in date seed oil: hydroxytyrosol, protocatechuic acid, tyrosol, gallic acid, caffeic acid, p-coumaric acid and oleuropein (26). 

In this paper the seed extracts of 14 native date varieties grown in Iran were screened for their antioxidant activities and polyphenol contents. 

## Experimental

*Plant material*

The seeds of 14 varieties (Shahani, Khasuei, Sayer, Zahedi, Shekar, Shahabi, Kabkab, Khenizi, Maktub, Kabkab dalaki 2, Shahabi 2, Majul, Goftar, Lasht), were procured from a local farm in Bushehr, Iran, at the beginning of the 2006 harvest season. The shape, size and color of each variety were completely different. Voucher specimens have been identified and deposited in the Herbarium of the Agricultural Research and Natural Resource Center of Bushehr province, Bushehr, Iran. They were dispatched by airplane to Tehran University of Medical Sciences. Mature fruits of uniform size, free of physical damage and injury from insects and fungal infection, were selected and used for all experiments. Upon arrival at the laboratory, the samples (500 g portions) were packed in polyethylene bags, sealed and stored at 2-8°C until analysis. The seeds were washed to get rid of any adhering date flesh, and air-dried. Then, they were further dried at about 50°C for 4 h. Date pits of each variety were separately milled in a heavy-duty grinder to pass 1-2 mm screens and then preserved at 2-8°C until analysis.

*Extraction methods*

About 0.02 g of powdered date seeds was shaked with 5 mL of solvent in a glass tube at room temperature, two times for 30 min and then centrifuged. Water, methanol:water (50:50, v/v), methanol, DMSO and water:methanol:acetone: formic acid (20:40:40:0.1) (3) were used as the best solvents. The extraction was carried out using five different solvents to compare the antioxidant activities and the total phenolic contents of each extract. 

*Evaluation of antioxidant activity using FRAP method*

The FRAP (ferric reducing antioxidant power assay) procedure described by Benzie and Strain was followed (27). The principle of this method is the reduction of a ferric-tripyridyl triazine complex to its colored ferrous form in the presence of antioxidants. Briefly, the FRAP reagent contained 5 mL of 10 mmol/L solution of TPTZ (2,4,6-tripyridyl-s-triazine) in 40 mmol/L HCL plus 5 mL of a 20 mmol/L solution of FeCl_3_ and 50 mL of a 0.3 mol/L acetate buffer solution, pH 3.6 which was prepared freshly and warmed at 37˚C. Aliquots of 50 μL extract were mixed with 1.5 mL FRAP reagent and after incubation at 37˚C for 10 min, the absorbance of the reaction mixture was measured at 593 nm. 

For construction of the calibration curve, five concentrations of FeSO_4_, 7H_2_O (1000, 750, 500, 250, 125 μmol ∕ L) were used and the absorbance values were measured as for sample solutions. The antioxidant activities were expressed as the concentration of antioxidants having a ferric reducing ability equivalent to that of 1 mmol/L FeSO_4_.

*Measurement of the total phenolic contents*

Total phenolics were determined colorimetrically using Folin-Ciocalteu reagent as described by Velioglu et al. (28) with slight modifications. The prepared extract (200 μL) was mixed with 1.5 mL of Folin-Ciocaltue reagent (previously diluted 10 fold with distilled water) and allowed to stand at 200°C for 5 min. A 1.5 mL sodium bicarbonate solution (60 g/L) was added to the mixture. After 90 min at 22°C, the absorbance was measured at 725 nm using a UV spectrophotometer (Pharmacia Biotech). The total phenolics were quantified by the calibration curve obtained from measuring the absorbance of a known concentration of gallic acid (GA) standard (20-150 mg/Lit). The concentrations were expressed as equivalent milligrams of gallic acid (GA) per 100 g dry plant.

*Statistical analysis*

The values were reported as mean ± SD. One-way ANOVA and Tukey post-hoc multicomparison tests were used for the analyses.

## Results and Discussion

To compare the effects of the extraction methods on antioxidant activities and total phenolic contents in 14 varieties of date seed, five different solvents were used (Table 1, 2). All the extracts had considerable antioxidant activities from the equivalent of 13.13 mmol of FeII/100 g dry plant in seeds of the Lasht variety to 37.42 mmol of FeII/100 g dry plant in Zahedi variety. All the extracts contained considerable phenolic contents from the equivalent of 1260 mg GA/100 g dry plant in seeds of the Shahabi variety to 3541 mg GA/100 g dry plant in Zahedi variety .

**Table 1:** Antioxidant activities of 14 varieties of date seed extracts using 5 different solvents (as mmol Fe^II^ /100 g dry plant).

** Solvent**	Water ^a^	Methanol (50%) ^a^	Methanol ^a^	Formic acid ^a,b^	DMSO ^a^
**Variety**					
Shahani	12.89 ± 0.33	9.77 ± 0.63	13.66 ± 0.50	26.06 ± 0.15	23.58 ± 0.39
Khasuei	13.45 ± 0.33	11.5 ± 0.13	14.06 ± 0.26	27.31 ± 0.34	33.64 ± 0.34
Sayer	11.70 ± 0.63	8.83 ± 0.33	13.29 ± 0.13	12 ± 0.53	19.62 ± 0.09
Zahedi	8.33 ± 0.18	14.41 ± 0.32	15.66 ± 0.28	23.54 ± 0.34	37.41 ± 0.47
Shekar	10.81 ± 0.46	8.52 ± 0.53	8.16 ± 0.21	10.16 ± 0.40	19.33 ± 0.45
Shahabi	9.29 ± 0.34	6.85 ± 0.09	7.81 ± 0.13	23.75 ± 0.36	13.29 ± 0.54
Kabkab	19.02 ± 0.54	26.02 ± 0.85	18.85 ± 2.42	28.64 ± 0.61	30.85 ± 0.44
Khenizi	9.93 ± 0.26	6.77 ± 0.43	17.458 ± 0.347	12.54 ± 0.31	23.62 ± 0.66
Maktub	6.54 ± 0.34	9.45 ± 0.5	15.43 ± 0.42	20.35 ± 0.09	21.29 ± 1.00
Kabkab dalaki 2	11.18 ± 0.36	21.54 ± 0.82	18.47 ± 0.58	28.5 ± 0.62	33.97 ± 0.09
Shahabi 2	4.64 ± 0.51	28.83 ± 0.68	18.75 ± 0.34	18.68 ± 0.56	21.89 ± 0.40
Majul	7.60 ± 0.34	8.91 ± 0.26	9.72 ± 0.15	13.27 ± 0.31	14.06 ± 0.32
Goftar	4.52 ± 0.16	4.34 ± 0.16	5.83 ± 0.15	7.01 ± 0.16	14.88 ± 0.21
Lasht	9.14 ± 0.16	6.16 ± 1.09	17.75 ± 0.46	14.10 ± 0.29	13.12 ± 0.34

^a^ Data are expressed as mean ± SD. Each sample was analyzed three times.

^b ^A mixture of water:methanol:acetone:formic acid (20:40:40:0.1).

**Table 2:** Total phenol contents of 14 varieties of date seed extracts using 5 different solvents (as mg GA/ 100 g dry plant).

** Solvent**	Water ^a^	Methanol (50%) ^a^	Methanol ^a^	Formic acid ^a,b^	DMSO ^a^
**Variety**					
Shahani	968 ± 40.41	838 ± 45.09	961 ± 35.11	2134 ± 45.82	3498 ± 25.16
Khasuei	1021 ± 37.78	898 ± 25.16	1341 ± 65.06	2858 ± 55.07	3378 ± 25.16
Sayer	1201 ± 100.16	1044 ± 79.37	1671 ± 45.09	854 ± 62.45	2088 ± 115.90
Zahedi	1161 ± 104.08	1431 ± 95.04	1448 ± 41.63	1744 ± 90.00	3541 ± 40.41
Shekar	817 ± 46.13	733 ± 26.23	812 ± 22.035	935 ± 50.64	1619 ± 33.40
Shahabi	873 ± 22.27	667 ± 32.33	812 ± 25.71	992 ± 37.80	1260 ± 28.37
Kabkab	1694 ± 125.3	1614 ± 55.67	1374 ± 70.00	1894 ± 110.00	2188 ± 15.27
Khenizi	954 ± 85.44	1681 ± 133.16	1581 ± 86.21	2724 ± 175.78	2041 ± 143.64
Maktub	961 ± 65.06	2624 ± 130.76	3284 ± 10.14	2028 ± 70.94	2438 ± 77.67
Kabkab dalaki2	1404 ± 13.52	2838 ± 127.14	2548 ± 75.71	3658 ± 325.32	2941 ± 56.86
Shahabi 2	681 ± 83.26	1608 ± 15.27	2021 ± 47.25	2048 ± 30.55	2078 ± 40.41
Majul	1044 ± 45.82	1161 ± 45.09	1258 ± 35.11	1831 ± 55.07	2108 ± 65.06
Goftar	441 ± 42.14	381 ± 36.66	459 ± 44.06	1103 ± 22.03	1616 ± 31.07
Lasht	971 ± 55.06	2404 ± 75.49	1741 ± 80.08	2454 ± 72.11	1741 ± 25.16

^a ^Data are expressed as mean ± SD. Each sample was analyzed three times.

^b^ A mixture of water:methanol:acetone:formic acid (20:40:40:0.1).

In previous investigations of *Phoenix dactylifera *L., Arecacea varieties, evaluation of the antioxidant activity and the presence of polyphenol compounds were reported (29). Antioxidant activities of different extracts of the edible portion of date fruit according to the β-carotene bleaching method ranged from 9.28% to 75.96%. The activity of petroleum ether seed extract was 8.19% and the total phenolic content was found to be 1416 mg of GA/100 g dry weight (29). In two different researches, antioxidant effects of the edible portion of hydro-alcoholic extracts of Kabkab and Zahedi varieties were reported as 11.7 and 19.1 (30), and 1510 and 1400 μmol Fe^II^/100 g dry plant (3). Also their total phenol contents were reported as 3250 and 4370 (30), and 200, 170 (3) mg GA/100 g dry plant. It seems that this variation was related to different method of extraction.

In another study, the flavonoid glycoside and procyanidin compositions of date were investigated and 19 flavonoid glycosides of luteolin, quercetin and apigenin were also identified (8). Besbes et al had investigated the quality and oxidative stability of the date seed oils from two date palm cultivators, Deglet Nour and Allig, during storage. Total phenolic contents were reported by them to be for them about 220-520 vg/g oil respectively and the results showed that the date seed oil could be easily stored (31). 

In this research, the antioxidant activities and phenolic contents of 14 date seed varieties were determined representing 37.42 mmol Fe^II^/100 g dry plant as the highest activity, and 3658 mg GA/100 g dry plant as the highest phenolic content in date samples compared to the previous works (29).

Significant differences (P < 0.001) existed among different solvents used, with some exception (Figures 1 and 2). Extraction by DMSO gave the highest antioxidant activity and total phenolic content, whereas water and methanol:water (50:50, v/v) resulted in the lowest values. The results showed that most of the potent antioxidant and phenolic compounds in the seeds of 14 date varieties were soluble in DMSO.

Although there was a relationship between the presence of high amounts of phenolic compounds, and the antioxidant effect of 14 varieties of date seeds in DMSO extracts (R^2^ = 0.791), there are some reports on the role of other compounds in this activity. The oleic acid contents, as a major antioxidant component of the date seeds varies from 41.1 to 58.8%, suggests that the seeds of date could be used as a source of oleic acid (4, 32). 

Date seeds have previously been examined for extractable high value-added components for incorporation into functional foods (33). The results showed that date seeds contained large quantities of fiber, and possibly resistant starch, which may have potential health benefits. 

Also in some other studies, the antioxidant effects of *Phoenix dactylifera *L. seeds were investigated. However, different methods were used for the studies, thus the results are not directly comparable.

As mentioned before, *Phoenix dactylifera *L., seeds could easily be conserved and used in cosmetics, pharmaceuticals and food products. Therefore, the potency of these extracts could provide a chemical basis for some of the health benefits claimed for *Phoenix dactylifera *L. seeds in folk medicine. Further studies are necessary to assess the potential of their components as effective natural remedies. 
